# 

**Published:** 1882-09

**Authors:** 


### CONTENTS.

### COMMUNICATIONS.

On the Causes of Dental Caries ..... 417
Aristocracy of Professions..... 420
American Dental Association ..... 423
Kentucky State Dental Association..... 448

### SELECTIONS.

Eucalyptus ..... 453
Zinc vs. Babbitt Metal ..... 456
Hemorrhage and Gangrene from Carious Tooth..... 457
Dental Tumor of Jaw..... 458
How to Apply the Soda Remedy in Burns and Scalds..... 459
Cautery..... 460
A Suggestion for the Laboratory..... 461
New Method of Preparing the Spinal Cord for Microscopic Sections.. 461
Otalgia from Reflex Irritation..... 461
The Administration of Chloroform..... 463

### CORRESPONDENCE.

Poulticing ..... 465

### EDITORIAL.

"Sanguinary Calculus?"..... 466
To be Resumed—The Mad River Dental Society..... 467
Obituary ..... 468

